# The need for sustainability and alignment of future support for National Immunization Technical Advisory Groups (NITAGs) in low and middle-income countries

**DOI:** 10.1080/21645515.2018.1444321

**Published:** 2018-03-26

**Authors:** Natasha Howard, Sadie Bell, Helen Walls, Laurence Blanchard, Logan Brenzel, Mark Jit, Sandra Mounier-Jack

**Affiliations:** a London School of Hygiene and Tropical Medicine, Department of Global Health and Development, Tavistock Place, London, UK; b Bill and Melinda Gates Foundation, Seattle, WA, USA; c London School of Hygiene and Tropical Medicine, Department of Infectious Disease Epidemiology, Keppel St, London, UK

**Keywords:** low and middle-income countries, NITAGs, vaccination, vaccine decision-making

## Abstract

National Immunisation Technical Advisory Groups (NITAGs) provide independent guidance to health ministries to support evidence-based and nationally relevant immunisation decisions. We examined NITAGs' value, sustainability, and need for support in low and middle-income countries, drawing from a mixed-methods study including 130 global and national-level key informant interviews. NITAGs were particularly valued for providing independent and nationally owned evidence-based decision-making (EBDM), but needed to be integrated within national processes to effectively balance independence and influence. Participants agreed that most NITAGs, being relatively new, would need developmental and strengthening support for at least a decade. While national governments could support NITAG functioning, external support is likely needed for requisite capacity building. This might come from Gavi mechanisms and WHO, but would require alignment among stakeholders to be effective.

## Introduction

The Global Vaccine Action Plan 2011–2020 called for all countries to establish or have access to a National Immunization Technical Advisory Group (NITAG) by 2020.^1^^,^^2^ NITAGs aim to provide independent technical guidance to national policy-makers and program managers to support evidence usage in immunization policy-making and programming.^3^ The SIVAC Initiative (2008–2017) provided technical support for establishing and strengthening NITAGs.^2^^,^^4^ By late 2016, 125 of 194 World Health Organization (WHO) member states reported having a NITAG, 120 (96%) of which were officially legislated – including 22 low-income and 69 middle-income countries.^5^ However, only 90 (46%) reported a functional NITAG, referring to the institutionalization and sustainability of this evidence-informed mechanism[5]. As a global immunization priority and marker of countries' commitment to immunization, NITAGs play an important role in ensuring value-for-money in immunization. However, despite NITAGs' potential importance in strengthening national decision-making, little independent research examines their institutional sustainability.^6-9^ With the end of the SIVAC project, it is useful to examine how these country-level efforts can be supported and sustained.

We consider NITAGs' value, sustainability, and need for continued financial and technical support, drawing from a mixed-methods study that included: (i) 45 global and 85 national-level key informant interviews in 25 countries; (ii) reviews of literature, administrative documents, and technical reports; and (iii) semi-structured NITAG meeting observation.^5^ Interviewees and countries were anonymized to protect confidentiality. The London School of Hygiene and Tropical Medicine research ethics committee provided approval (reference 12036).

### NITAGs' value

NITAGs were particularly valued for providing an independent evidence-based decision-making (EBDM) process that was nationally owned. Interviewees noted that NITAG decision-making appeared more independent and evidence-based than decisions by other national bodies, e.g. Inter-agency Coordinating Committees (ICCs), and NITAGs potentially balanced the influence of industry lobbyists. NITAGs increased country ownership of vaccine decision-making due to members' professional credibility and emphasis on using evidence, particularly local data, to support decision-making. For example, Senegal's NITAG recommended birth-dose Hepatitis B administration within 72 rather than 24 hours of birth, as most births occurred at home.^5^ Interviewees indicated that NITAGs added value to immunization programs. However, some expressed concerns that, as most countries have already decided whether to introduce many of the new vaccines available and schedules are becoming increasingly complex, necessary expertise may be beyond the capacities of most LMIC NITAGs and require broadening NITAGs' longer-term scope.

“The role of NITAGs should not be limited to the consideration of recommendations for the introduction of new vaccines, but should extend to devising strategies for optimizing the use of existing vaccines and strengthening national immunization programs.” (Country#0–1)

To maintain value, NITAGs must be integrated within broader health system decision-making in ways that maintain a balance between independence and influence. The initial NITAG model supported in LMICs, based on the WHO/SAGE approach of assessing epidemiological evidence, must incorporate economic/affordability aspects more routinely. For example, NITAG deliberations infrequently addressed affordability and sustainability of multiple vaccine introductions, a particular challenge for countries transitioning from Gavi support.^5^ Capacity to consider efficiency and financial sustainability was noted as critical for countries.

Perceived value by the global health community also affects NITAG sustainability. NITAGs were initially undervalued at global-level due to perceptions of weak country-level capacity, that decision-making should follow WHO/SAGE recommendations, and/or that NITAGs might delay vaccine introductions. Interviewees argued that EBDM should be tailored to individual country needs and capacities. Additionally, there was little consensus on how NITAGs could better complement other decision-making bodies, e.g. ICC, Health Technology Assessment, and polio national certification committees. Several noted that country-level separation between technical bodies reflected siloed funding streams. Delineation between country, regional, and global vaccine decision-making bodies showed some functional overlap and duplication. However, most interviewees supported country-level EBDM, indicating that over time governments and the global health community recognized the value of NITAGs in EBDM. Nevertheless, country interviewees advocated that NITAG support be independent from those external partners with direct interest in introducing specific vaccines, enabling countries to ‘push back'.

### NITAGs' sustainability and support needs

Most LMIC NITAGs faced some risks to sustainability, as proper functioning required time, funding, and staffing commitments. NITAGs that were rapidly set-up as volunteer committees without dedicated budgets were able to meet as schedules permitted, but had no funding (e.g. to support data collection and analysis, trainings, country-specific studies). Thus, those NITAGs that lacked allocated secretariat support, had no dedicated office space or transport funds, or lacked training in EBDM, could continue to exist indefinitely, but not fully functionally, and quality of deliberations were affected. Such NITAGs were more likely to rely on expert opinion for decision-making and were less effective than NITAGs that received regular financial and technical support from government or partners.

“NITAGs are valuable and necessary, but we will continue to need longer-term support to be effective, e.g. technical, funding, facilitation of networking, and develop collaborations with other NITAGs.” (Country#10–1)

Interviewees acknowledged that focus was needed on NITAG sustainability as they became more functional. A clear need identified by most NITAGs was support for economic and budget impact analysis. Conducting cost-effectiveness analyses to support decision-making, particularly with lack of global agreement on cost-effectiveness thresholds, was recognized as crucial but technically challenging for NITAGs. Some suggested developing a menu of modelling tools for countries, potentially including simple decision-making support models.

### Future NITAG support

While NITAGs have been supported globally by the SIVAC initiative (2008–2017), WHO headquarters and regional offices, Gavi the Vaccine Alliance, US-CDC, continued funding is challenging for many NITAGs now that SIVAC has ended. Interviewees agreed that most LMIC NITAGs, being relatively new, would need financial and technical support for the longer term. Most interviewees indicated that governments should provide NITAG funding, as this appeared most reliable so long as NITAGs remained able to make independent decisions. However, such funding might be insufficient to support NITAG capacity-building initiatives, such as training and peer visits. Other funders (e.g. Gavi, WHO) may thus need to support these country-level efforts.

NITAG support could come from Gavi Health System Strengthening (HSS) funding, though between 2012 and 2015 only Nigeria's NITAG appeared to receive some HSS support. Thus, Gavi targeted country assistance mechanisms may be another vehicle for needed NITAG strengthening support. WHO has been a long-standing supporter of NITAGs. While many NITAGs receive some WHO support (e.g. meeting rooms, travel expenses, training), amounts were expected to remain limited. WHO does support the Global NITAG Network (GNN), which facilitates cross-country exchange and peer-learning[8]. Some interviewees suggested that Regional TAGs (R-TAGs) could provide additional technical support to NITAGs, given some restructuring to allow country participants greater voice.

### Aligning support

Alignment between streams of support was noted as increasingly important. Over the years, numerous initiatives have supported decision-making, particularly for new vaccines. There was no agreement whether these had been complementary or had contributed holistically to strengthening decision-making processes. Interviewees noted that although many programs worked well together (e.g. SIVAC, ProVac, PATH) in the short-term, this appeared to be based on informal cooperation with no systemic approach taken to strengthening local capacity. Some suggested focusing regionally and/or devising support mechanisms adapted to country needs.

“Doing everything globally to build capacity globally, is not empowering. It doesn't build regions, it doesn't build strength in that sense…” (Global#54)

Since 2009, WHO/Unicef has included presence of a NITAG and six NITAG functionality process indicators in the Joint Reporting Form[9]. Another important step towards aligning NITAG support among stakeholders was the recent requirement by Gavi to include NITAG recommendations in funding applications. However, some interviewees expressed concern that reliance on global support could morph into bureaucratic ‘box-ticking’ rather than genuine strengthening of national EBDM capacity. The GNN, as a specific forum for NITAG peer-support and advocacy[8], could play a role in aligning organizational processes. To be effective, NITAG capacity strengthening support must be available long-term rather than as time-limited project funding.

“I think there is a need for support, not just setting them up but training the committee members to really be able to interpret this evidence.” (Global#36)

## Conclusions

What might an effective future support model look like? Having seen the potential value of NITAGs, many LMICs are moving forward with NITAG development. However, the risk remains that without sustainable funding and technical support, NITAG capacity for independent immunization-related deliberation will be limited. All evidence indicates that technical and funding support to LMIC NITAGs is needed for many more years. While daily running costs are probably best met through national budgets, data are limited on what it costs to operate a NITAG effectively. More research is needed to establish and support the sustainability of this important mechanism, while new mechanisms are needed to provide the ongoing EBDM technical guidance that was the most valued aspect of previous support.
